# Supplementary material for: Post-surgical adhesions are triggered by calcium-dependent membrane bridges between mesothelial surfaces
Source: Nat Commun. 2020 Jun 17;11:3068. doi: 10.1038/s41467-020-16893-3 (PMC7299976; doi:10.1038/s41467-020-16893-3)
Supplement: Supplementary file 1 — Supplementary information [file 41467_2020_16893_MOESM1_ESM.pdf]

Post-surgical adhesions are triggered by calcium-dependent membrane bridges between mesothelial surfaces

Adrian Fischer[ξ], Tim Koopmans[ξ], Pushkar Ramesh, Simon Christ, Maximilian Strunz, Juliane Wannemacher, Michaela Aichler, Annette Feuchtinger, Axel Walch, Meshal Ansari, Fabian J. Theis, Kenji Schorpp, Kamyar Hadian, Philipp-Alexander Neuman, Herbert B. Schiller, and Yuval Rinkevich[*]

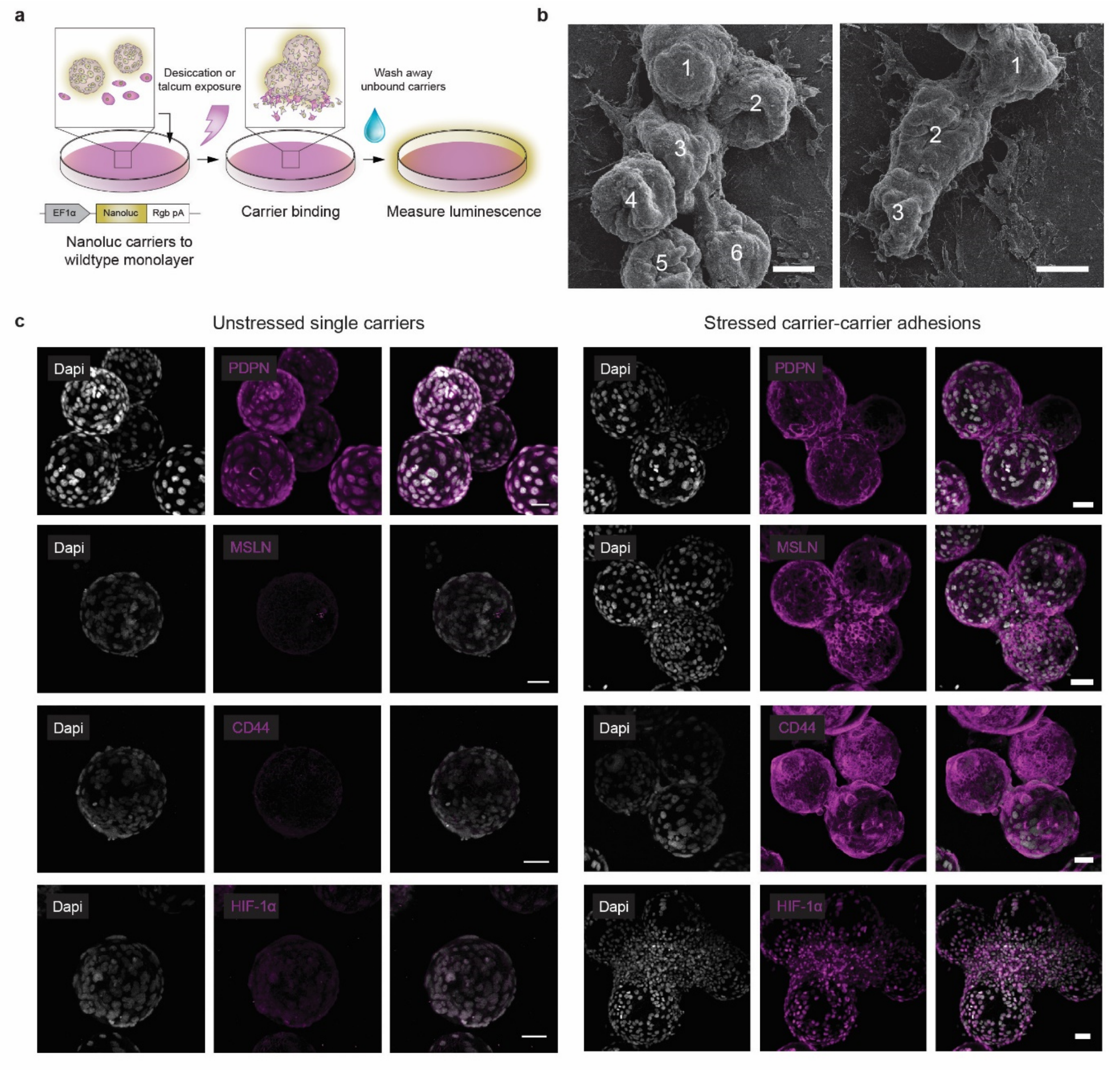

Supplementary Figure 1 – Carrier aggregates express the same characteristic adhesions proteins as in vivo adhesions. a, Overview of the nanoluciferase assay. b, Scanning electron microscopy image of clustered beads. Scale bar, 50 µm. c, Bead clustering induces biomarker expression in 3D whole mount stains, 2 days after stress. Scale bar, 50 µm.

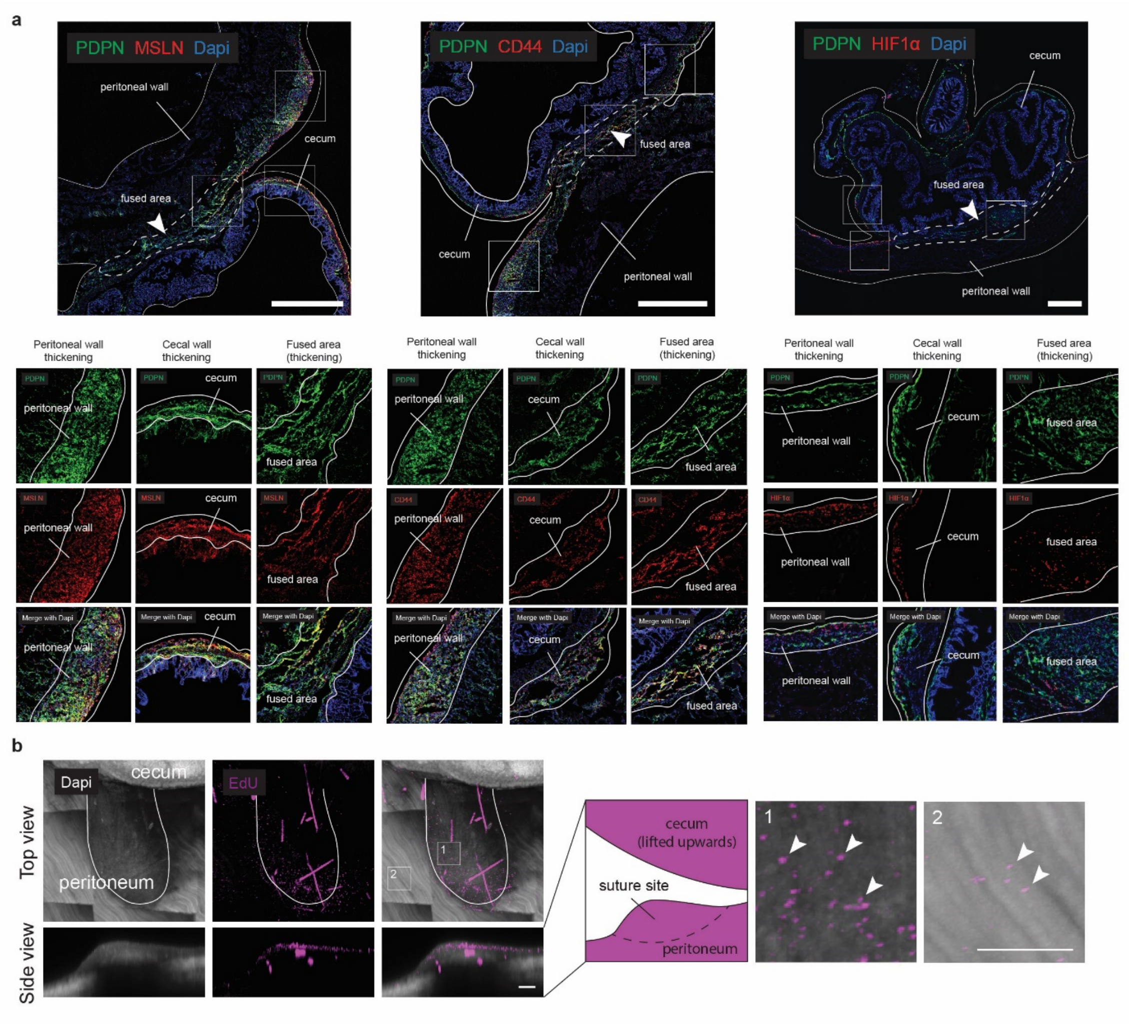

Supplementary Figure 2 – Carrier aggregates express the same characteristic adhesions proteins as in vivo adhesions. a, Adhesion tissue sections showing organ tethering, PDPN+ mesothelial thickening, and biomarker expression 5 days after injury. Scale bar, 1000 µm. b, Top and side view of whole mount stain showing EdU labelling on suture site and adjacent areas 5 days after adhesion injury. Scale bar, 500 µm.

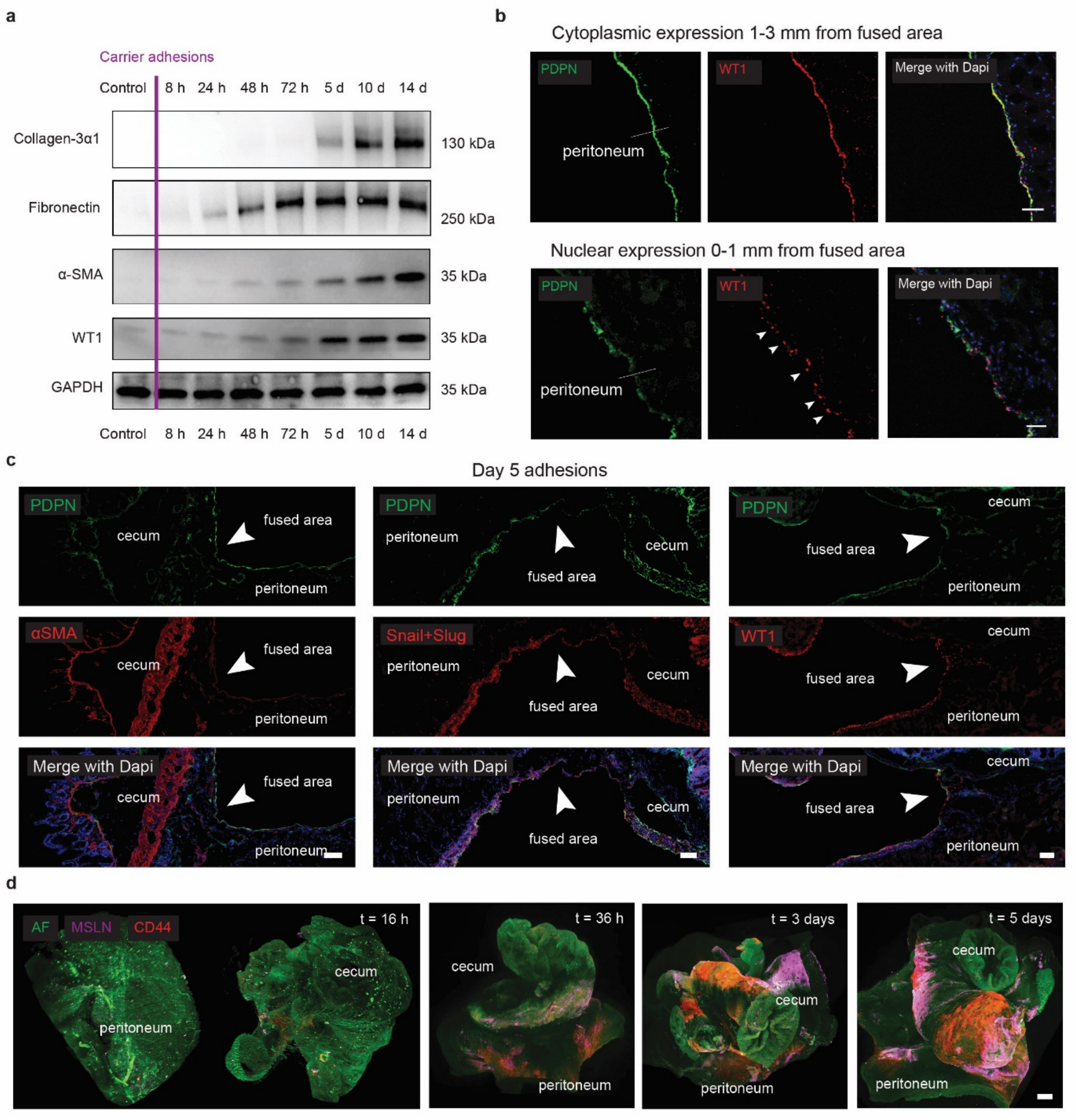

Supplementary Figure 3 – EMT marker expression develops only after adhesions have already formed. a, Representative immuno-blots of stressed and unstressed Met-5A cells after a 15 desiccation shock. GAPDH serves as loading control. b, Confocal image of adhesion peritoneal tissue sections showing cytosolic and nuclear WT1 staining far and close to the adhesion centre respectively, with active signalling (nuclear) close to the centre. Scale bar, 100 µm. c, Confocal images of adhesion tissue sections 5 days after injury, immuno-stained for mesenchyme and EMT markers. Scale bar, 50 µm. d, Whole-mount cleared light-sheet microscopy image of adhered organs showing increased MSLN and CD44 expression over time. AF, auto fluorescence. Scale bar, 100 µm.

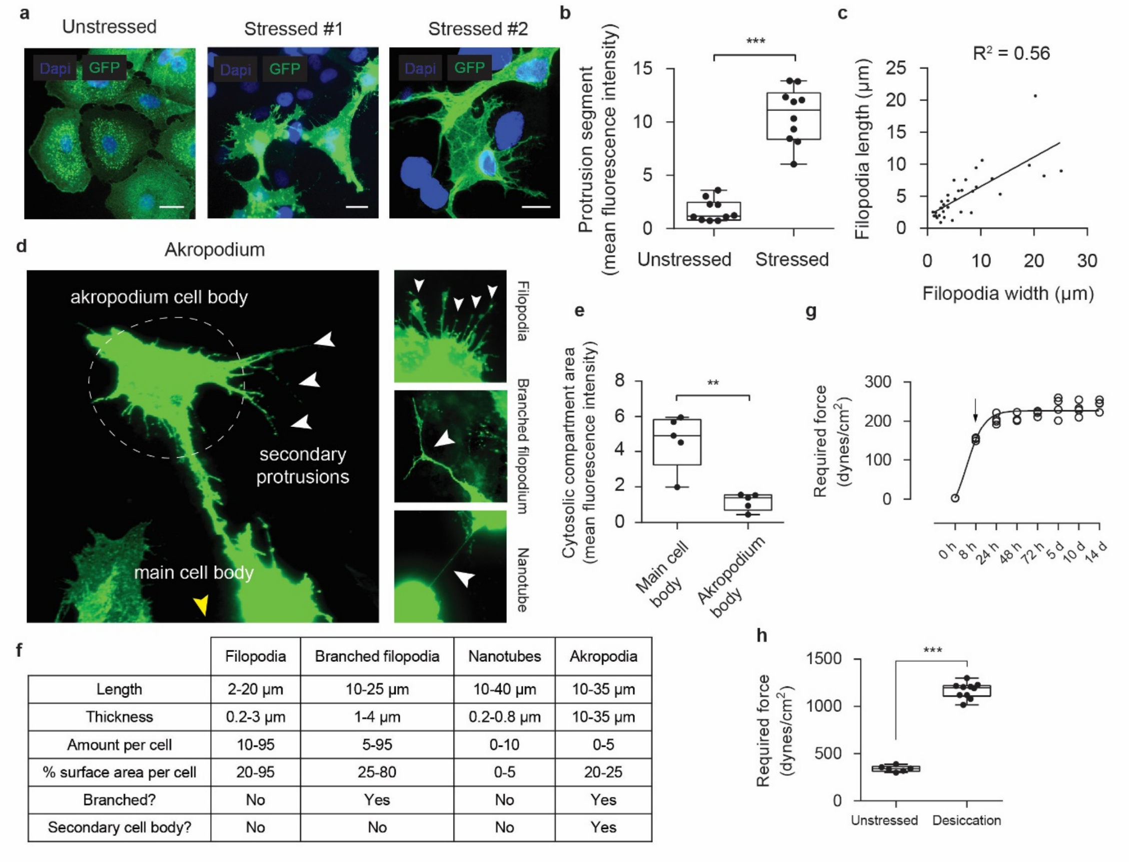

	Filopodia	Branched filopodia	Nanotubes	Akropodia
Length	2-20 µm	10-25 µm	10-40 µm	10-35 µm
Thickness	0.2-3 µm	1-4 µm	0.2-0.8 µm	10-35 µm
Amount per cell	10-95	5-95	0-10	0-5
% surface area per cell	20-95	25-80	0-5	20-25
Branched?	No	Yes	No	Yes
Secondary cell body?	No	No	No	Yes

Supplementary Figure 4 – Stressed mesothelia express a multitude of protrusions. a, Met-5A cells stably expressing membrane bound GFP, drastically changing morphology after desiccation shock, generating radial protrusions. b, Total surface area of protrusions in stressed and unstressed cells, 24 hours after a 15 min desiccation shock. Quantification derived from images, as in (a), performed through machine learning algorithms based on the 'advanced weka segmentation' Fiji plugin, employed to detect the total filopodial area in stressed mesothelia. Several filopodial types are present. *** $p < 0.001$, two tailed t-test. c, Distribution of total filopodial length and width in stressed Met-5A cells. d, Representative images showing the main protrusion types found in stressed Met-5A cells. e, Main and akropodial cell body, using machine learning as in (b). ** $p < 0.01$, two tailed t-test. f, Characteristics of different protrusions present on stressed mesothelia, measured using machine learning as in (b). g, Spinning disc data showing required forces to detach carrier clusters, various time points after a 15 min desiccation shock. h, Spinning disc data as in (g) of carrier-to-monolayer attachments. *** $p < 0.001$, two tailed t-test. Error bars represent standard error of the mean.

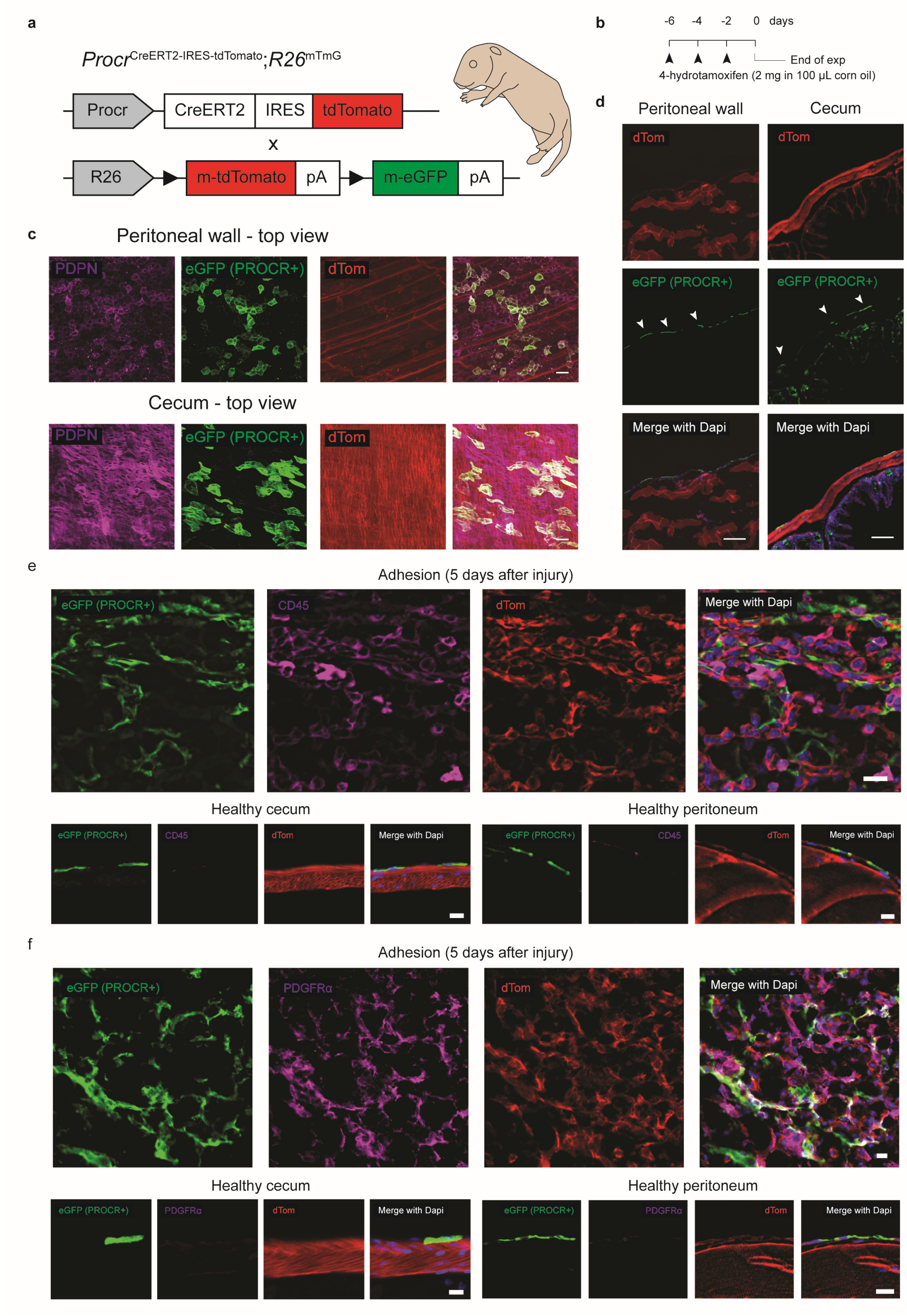

. **Supplementary Figure 5** – PROCR-Cre(ER) mice selectively label mesothelial cells. a, Overview of the PROCR-Cre(ER) transgene used. b, Tamoxifen regime to label PROCR+ mesothelium. c, Whole-mount confocal image of PROCR-Cre(ER) mice treated with tamoxifen, showing approximately 50% of labelled mesothelium on the peritoneal and cecal surface. Scale bar, 40 µm. d, Confocal image of peritoneal and cecal tissue sections after tamoxifen treatment, showing staining is limited to the surface. Scale bar, 50 µm. e, f,

Confocal image of tamoxifen-treated Procr$^{CreERT2\text{-}IRES\text{-}tdTomato}$;Rosa26^{mTmG} mouse adhesion tissue section, showing the adhesion core, 5 days after injury, and the corresponding healthy tissues. Co-labelled for the pan-immune marker CD45 (e) and the pan-fibroblast marker PDGFRα (f). Scale bar, 50 µm.

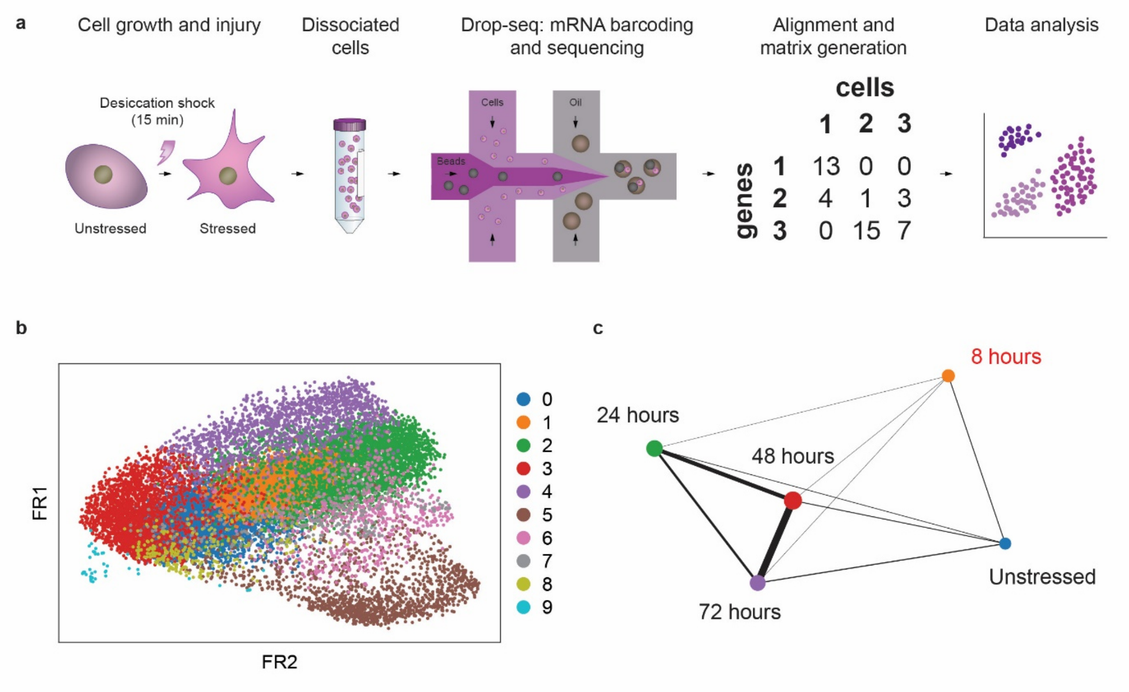

Supplementary Figure 6 – Single-cell RNAseq identifies actin and calcium pathways in stressed mesothelia. a, Overview of the Dropseq workflow. b, t-SNE visualization of >16.000 Met-5A cells coloured by cluster assignment. c, Partition based graph abstraction of the different experimental groups.

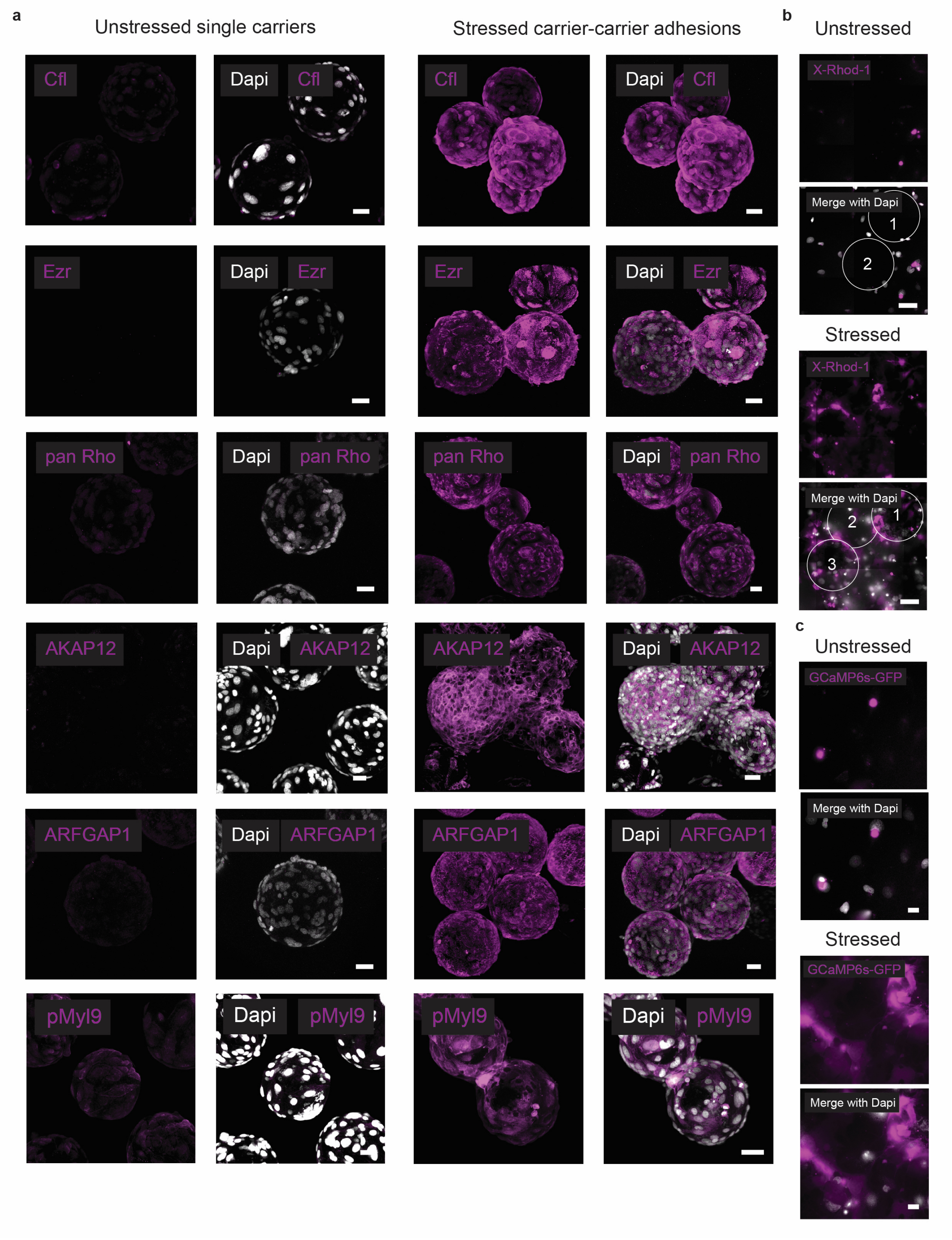

Supplementary Figure 7 – Stress adhesion markers are conserved across species. a, Confocal whole-mount image of carrier-carrier complexes 2 days after a 15 min desiccation shock, immuno-stained for core adhesion genes. Scale bar, 40 µm. b, Epi-fluorescent image (top view) of carrier-to-monolayer Met-5A cells 24 hours after desiccation, treated with the permeable calcium reporter X-Rhod-1. Scale bar, 150 µm. c, Epi-fluorescent

image (top view) of carrier-to-monolayer Met-5A cells expressing the calcium indicator GCaMP6s, 24 hours after desiccation. Scale bar, 150 µm.

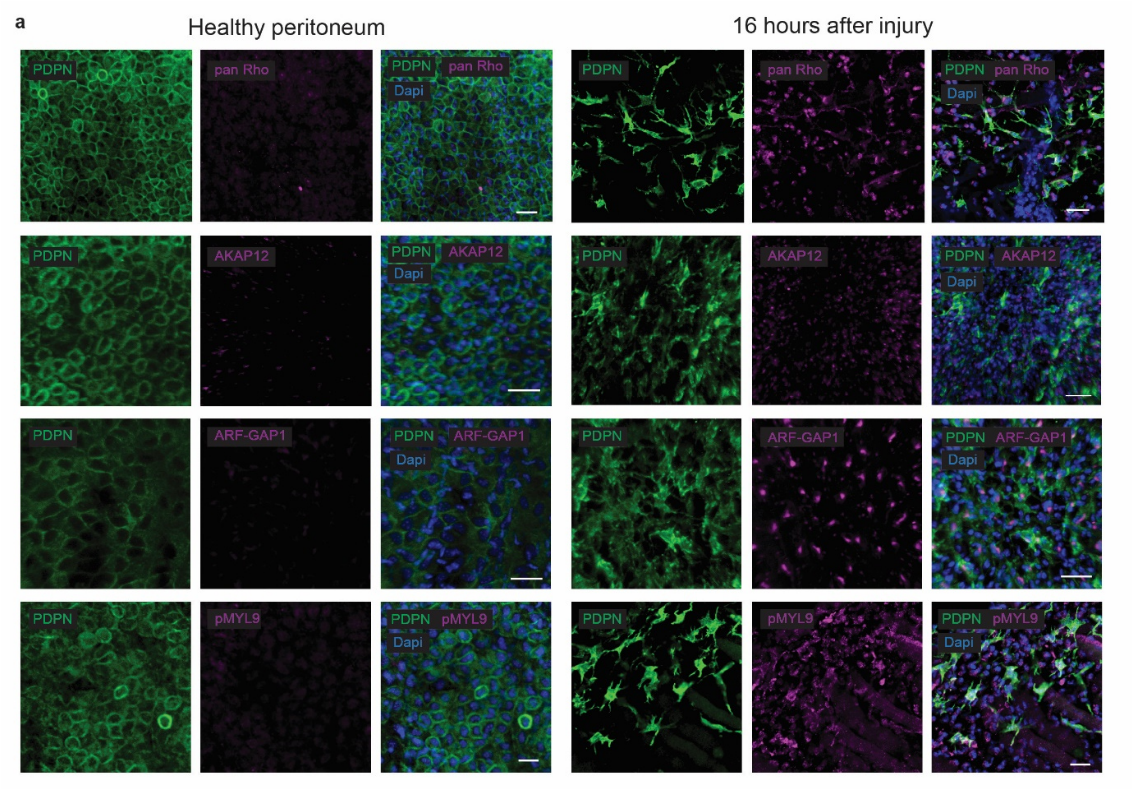

Supplementary Figure 8 – Stress adhesion markers are conserved across species. a, Confocal whole-mount image of PDPN+ peritoneal in vivo adhesions 16 hours after injury, immuno-stained for core adhesion genes. Scale bar, 40 µm.

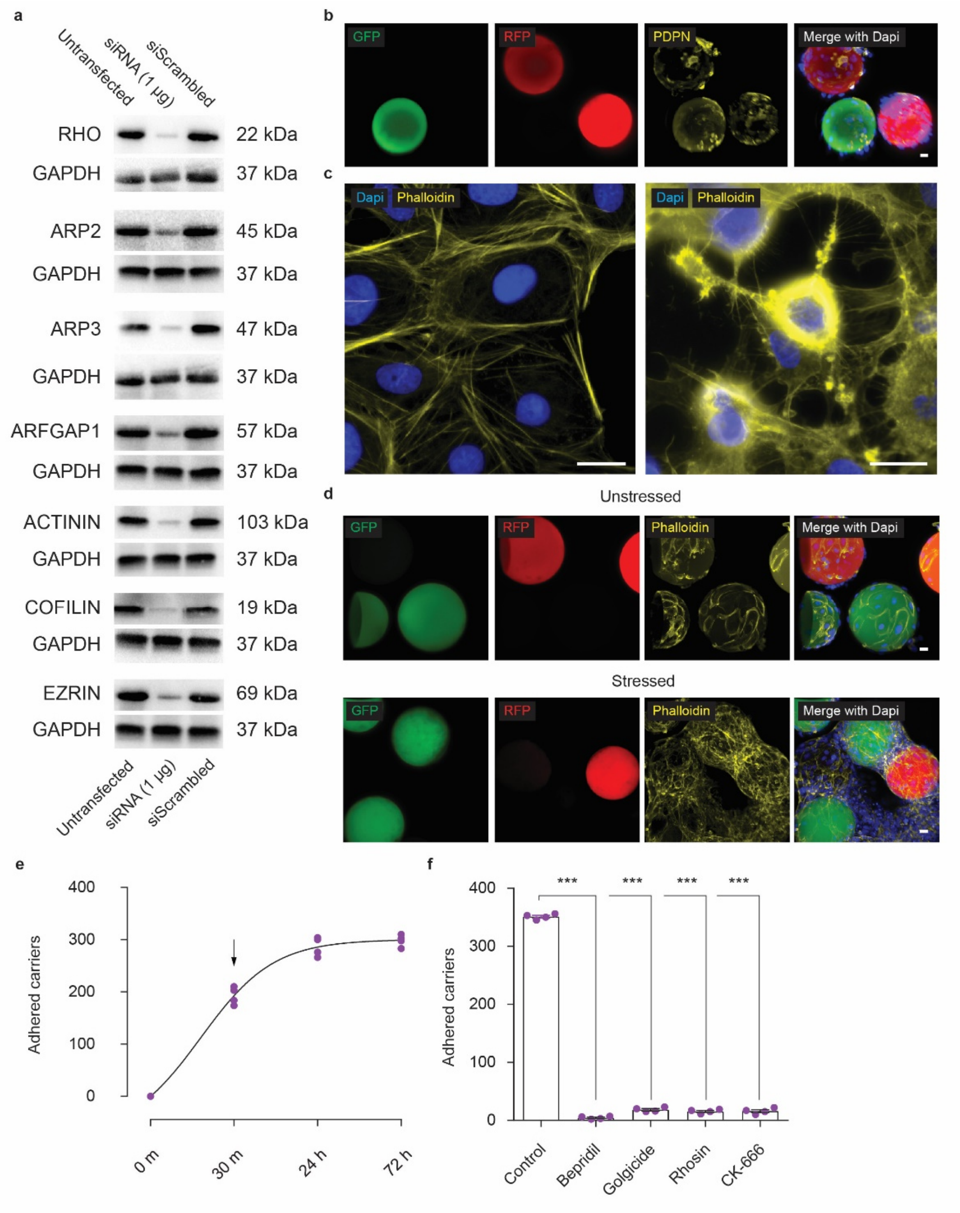

Supplementary Figure 9 – Primary abdominal mesothelium recapitulates adhesion biology. a, Representative immuno-blots of lysed Met-5A cells treated with small-interfering RNA against core adhesion genes. GAPDH serves as loading control. b, Confocal whole-mount image of PDPN+ primary abdominal mesothelium coated on beads soaked in fluorescent dye (GFP or RFP). Scale bar, 20 µm. c, Epi-fluorescent image of primary abdominal mesothelium cultured as a monolayer, 24 hours after a 15 min desiccation shock. Scale bar, 20 µm.

d, Confocal whole-mount image of PDPN+ primary abdominal mesothelium coated on beads soaked in fluorescent dye (GFP or RFP), 24 hours after a 15 min desiccation shock. Scale bar, 20 µm. e, Adhesion kinetics of primary mesothelium-covered beads adhering to a monolayer after a 15 min desiccation shock. f, Number of adhered carriers to a monolayer 24 hours after a 15 min desiccation shock, treated with different small molecules (10 µM) targeting core adhesion genes. *** $p < 0.001$, two tailed t-test. Error bars represent standard error of the mean.

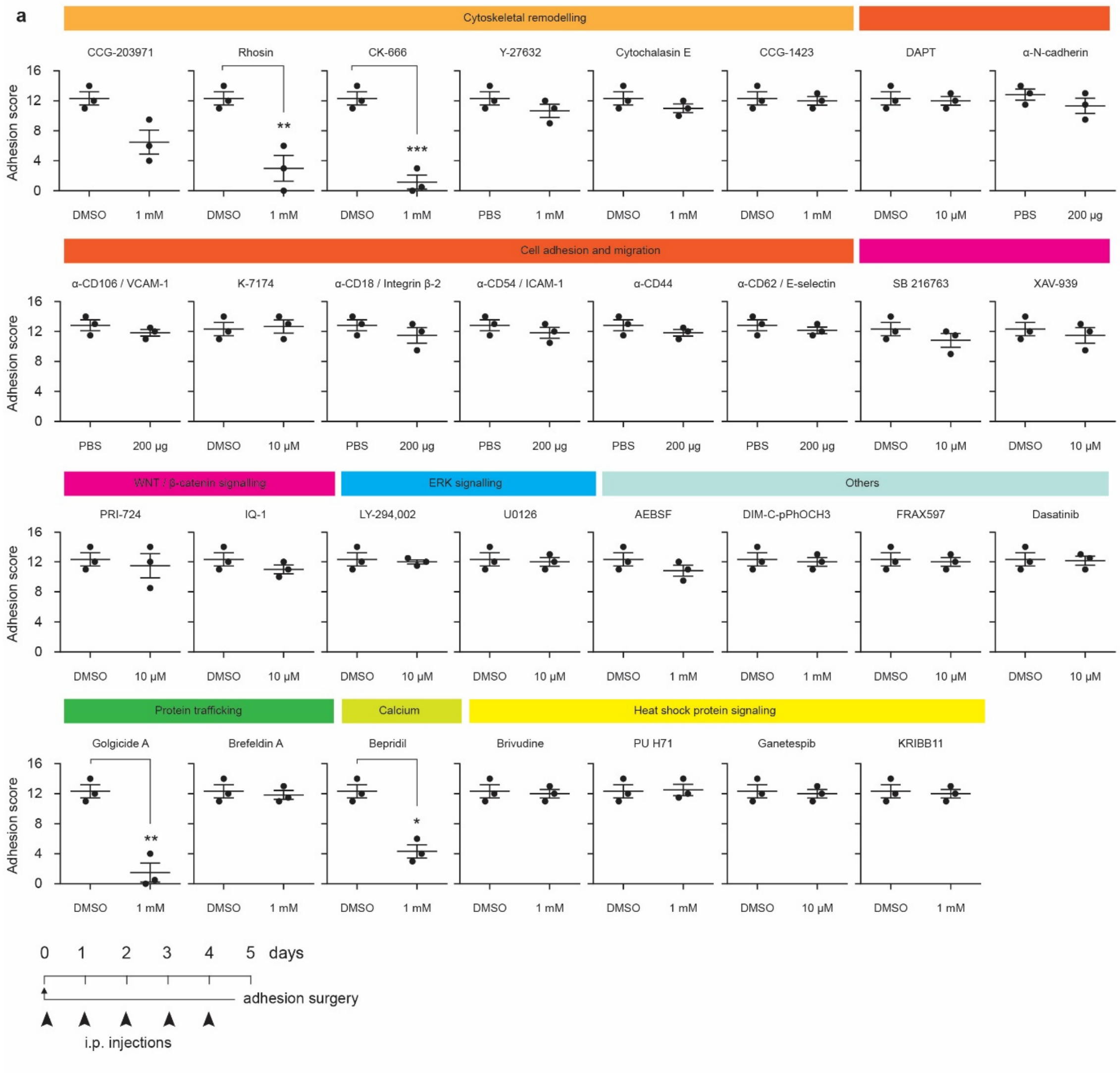

Supplementary Figure 10 – Small molecule screen highlights selectivity of actin and calcium pathways. a, Adhesion score 5 days after injury, of mice treated with small-molecule compounds injected intraperitoneally daily for 5 days. * $p < 0.05$, ** $p < 0.01$, *** $p < 0.001$, two-tailed Mann-Whitney. Error bars represent standard error of the mean.

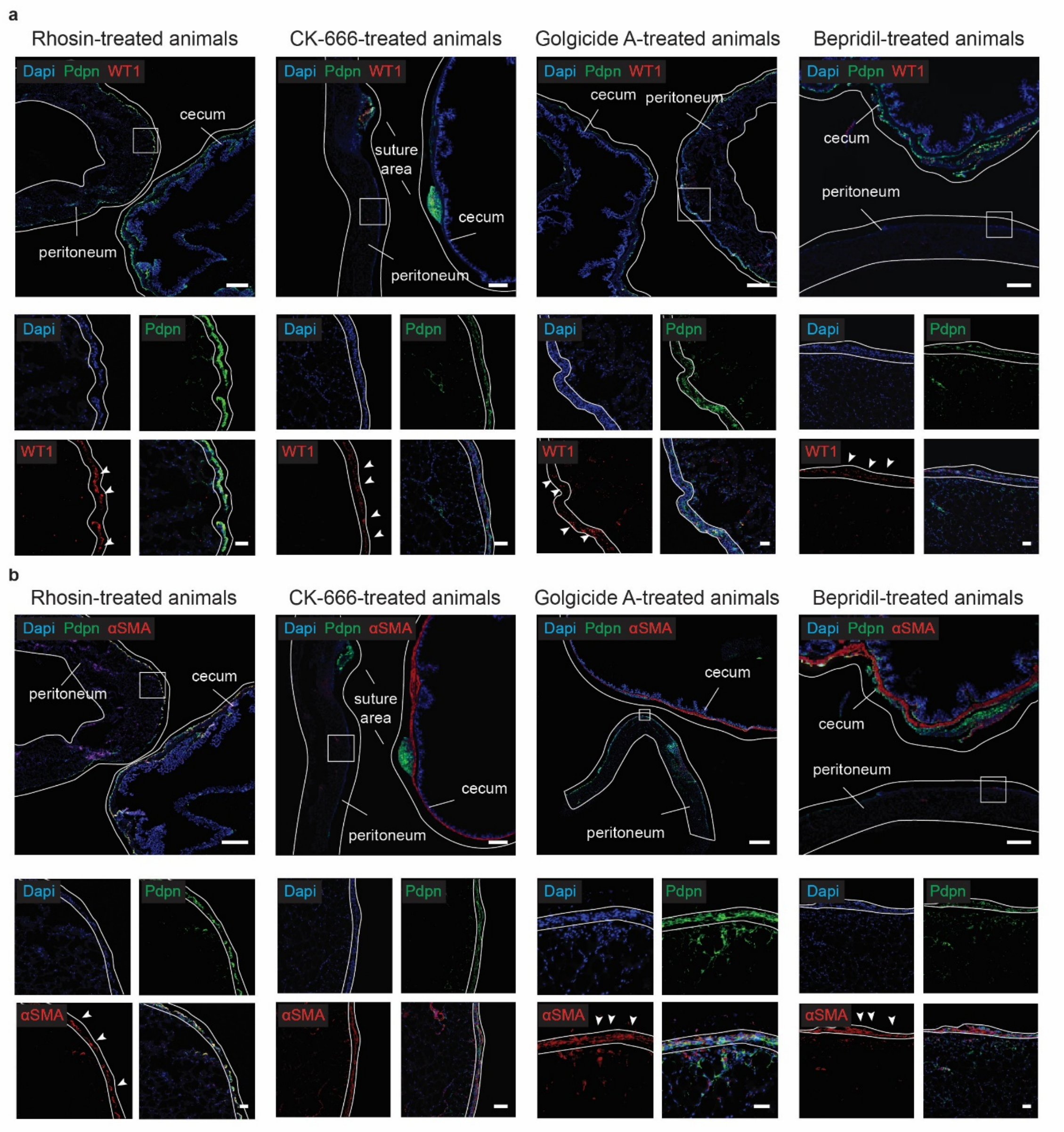

Supplementary Figure 11 – Core adhesion genes drive tethering, but not EMT or thickening. a and b, Adhesion tissue sections treated with small molecules targeted against core adhesion genes, immuno-stained for EMT and mesenchymal markers. Scale bar, 500 µm (overview) and 50 µm (inlet).

Supplementary Video 1 – Side view live imaging of Hoechst labelled Met-5A cells (carrier-to-monolayer)

Supplementary Video 2 – 3D reflectance confocal imaging of stressed mesothelia seeded on carriers

Supplementary Video 3 – Stressed membranous GFP/mCherry labelled Met-5A cells grown on a monolayer

Supplementary Video 4 – Independent filopodial movement on akropodium in LifeAct-mCherry stressed Met-5A cells seeded on carriers

Supplementary Video 5 – Time lapse of peritoneal PROCR+ mesothelium 4-12 hours after injury

Supplementary Video 6 – Carrier aggregates seeded with stressed LifeAct-eGFP/mCherry Met-5A cells

Supplementary Table 1: Scoring table (cumulative value determines total adhesion score)

Nr. of attached sutures		Nr. of attached secondary organs		Total adhesive coverage		Attached colon?		Adhesion supported by gravity or mild pulling strength?	
Outcome	Score	Outcome	Score	Outcome	Score	Outcome	Score	Outcome	Score
0	0	0	0	0 cm^2	0	No	0	No	0
1	1.5	1	1	0-0.5 cm^2	1	Yes	3	Yes	3
2	3	2	2	0.5-1.0 cm^2	2				
		3	3	>1.0 cm^2	3				

Supplementary Table 2: Antibody list

PDPN (mouse)	Abcam, #ab11936	1:500
PDPN (human)	Abcam, #ab10288	1:500
MSLN	ABBIOTEC, #250519	1:200
HIF-1α	Abcam, #ab179483)	1:200
CD44 polyclonal	Abcam, #ab157107	1:200
CD44 monoclonal	Abcam, #ab119348	1:200
Fibronectin	Abcam, #ab23750	1:50
Collagen-3α1	Abcam, #ab7778	1:100
α-SMA	Abcam, #ab21027	1:200
WT1	Abcam, #ab89901	1:200
GAPDH	New England Biolabs, #2118S	1:2000
phosphoMYL9	Cell Signaling Technology, #3674	1:200
Cofilin	Abcam, #ab42824	1:200
Ezrin	Abcam, #ab4069	1:200
ARFGAP1	Abcam, #ab204405	1:200
pan Rho	Abcam, #ab40673	1:200
α-Actinin	Abcam, #ab18061	1:200
AKAP12	Invitrogen, #PA552281	1:200
ARP2	Santa Cruz, #sc-376698	1:200
ARP3	Santa Cruz, #sc-48344	1:200

Supplementary Table 3: Small molecule list

Echinomycin	Sigma Aldrich, #SML0477
Bepridil	Santa Cruz, #sc-202974A
Rhosin	R&D systems, #5003/10
CK-666	Abcam, #ab141231
Golgicide A	Sigma Aldrich, #G0923
CCG-203971	Sigma Aldrich, #SML1422
Y-27632	Sigma Aldrich, #Y0503
Cytochalasin E	Tebu-Bio, #170BIA-C1251-1
CCG-1423	Sigma Aldrich, #SML0987
DAPT	Sigma Aldrich, #D5942
K-7174	Enzo Life sciences, #BV-9478
SB 216763	Biomol, #Cay10010246
XAV-939	Biomol, #Cay13596
PRI-724	Biozol, #SEL-S8262
IQ-1	Biozol, #SEL-S8248
LY-294,002	Biozol, #S1105
U0126	Biozol, #S1102
AEBSF	Santa Cruz, #sc-202041
DIM-C-pPhOCH3	Sigma Aldrich, #D7946
FRAX597	Biozol, #S7272
Dasatinib	Biozol, #S1021
Brefeldin A	VWR, #A2138.0005
Brivudine	Sigma Aldrich, #B9647
PU H71	R&D systems, #3104/10
Ganetespib (STA-9090)	Enzo Life sciences, #BV-9459
KRIBB11	Merck, #385570

Supplementary Table 4: Blocking antibody list

Anti-N-cadherin	Bio X Cell, #BE0184
Anti-CD106 / VCAM	Bio X Cell, #BE0027
Anti-CD18 / Integrin β-2	Bio X Cell, #BE0009
Anti-CD54 / ICAM-1	Bio X Cell, #BE0020-1
Anti-CD44	Bio X Cell, #BE0039
Anti-CD62 / E-selectin	Bio X Cell, #BE0294

Supplementary Table 5: Plasmid list

piggyBac AF1
piggyBac AF14
piggyBac AF15
piggyBac AF23
piggyBac AF28
piggyBac AF29
piggyBac AF32
piggyBac AF33
piggyBac AF34
piggyBac AF50
piggyBac transposase helper
Lifeact-EGFP (Addgene, #54491)
Lifeact-mCherry (Addgene, #58470)

Supplementary Table 6: siRNA list

**Target**	**Company**	**Catalog number**
Control siRNA-A	Santa Cruz	sc-36869
Arp2 siRNA (h)	Santa Cruz	sc-29737
Arp3 siRNA (h)	Santa Cruz	sc-29739
α-Actinin-1 siRNA (h)	Santa Cruz	sc-43095
Ezrin siRNA (h)	Santa Cruz	sc-35349
ARFGAP1 siRNA (h)	Santa Cruz	sc-72529
Rho A/B/C siRNA (h)	Santa Cruz	sc-44076
Cofilin 1 siRNA (h)	Santa Cruz	sc-35078
