# Supplementary material for: Post-surgical adhesions are triggered by calcium-dependent membrane bridges between mesothelial surfaces
Source: Nat Commun. 2020 Jun 17;11:3068. doi: 10.1038/s41467-020-16893-3 (PMC7299976; doi:10.1038/s41467-020-16893-3)
Supplement: Supplementary file 2 — Description of Additional Supplementary Files [file 41467_2020_16893_MOESM2_ESM.pdf]

Title: Supplementary Video 1
Description: Side view live imaging of Hoechst labelled Met-5A cells (carrier-to-monolayer)

Title: Supplementary Video 2
Description: 3D reflectance confocal imaging of stressed mesothelia seeded on carriers

Title: Supplementary Video 3
Description: Stressed membranous GFP/mCherry labelled Met-5A cells grown on a monolayer

Title: Supplementary Video 4
Description: Independent filopodial movement on akropodium in LifeAct-mCherry stressed Met5A cells seeded on carriers

Title: Supplementary Video 5
Description: Time lapse of peritoneal PROCR+ mesothelium 4-12 hours after injury

Title: Supplementary Video 6
Description: Carrier aggregates seeded with stressed LifeAct-eGFP/mCherry Met-5A cells
