# Supplementary material for: Post-surgical adhesions are triggered by calcium-dependent membrane bridges between mesothelial surfaces
Source: Nat Commun. 2020 Jun 17;11:3068. doi: 10.1038/s41467-020-16893-3 (PMC7299976; doi:10.1038/s41467-020-16893-3)
Supplement: Supplementary file 9 — Reporting Summary [file 41467_2020_16893_MOESM9_ESM.pdf]

nature research

Corresponding author(s): Yuval Rinkevich

Last updated by author(s): May 4, 2020

Reporting Summary

Nature Research wishes to improve the reproducibility of the work that we publish. This form provides structure for consistency and transparency in reporting. For further information on Nature Research policies, see our Editorial Policies and the Editorial Policy Checklist.

Statistics

For all statistical analyses, confirm that the following items are present in the figure legend, table legend, main text, or Methods section.

n/a	Confirmed	
☐	☒	The exact sample size (*n*) for each experimental group/condition, given as a discrete number and unit of measurement
☐	☒	A statement on whether measurements were taken from distinct samples or whether the same sample was measured repeatedly
☐	☒	The statistical test(s) used AND whether they are one- or two-sided<br>*Only common tests should be described solely by name; describe more complex techniques in the Methods section.*
☐	☒	A description of all covariates tested
☐	☒	A description of any assumptions or corrections, such as tests of normality and adjustment for multiple comparisons
☐	☒	A full description of the statistical parameters including central tendency (e.g. means) or other basic estimates (e.g. regression coefficient) AND variation (e.g. standard deviation) or associated estimates of uncertainty (e.g. confidence intervals)
☐	☒	For null hypothesis testing, the test statistic (e.g. *F*, *t*, *r*) with confidence intervals, effect sizes, degrees of freedom and *P* value noted<br>*Give P values as exact values whenever suitable.*
☐	☒	For Bayesian analysis, information on the choice of priors and Markov chain Monte Carlo settings
☐	☒	For hierarchical and complex designs, identification of the appropriate level for tests and full reporting of outcomes
☐	☒	Estimates of effect sizes (e.g. Cohen's *d*, Pearson's *r*), indicating how they were calculated

Our web collection on statistics for biologists contains articles on many of the points above.

Software and code

Policy information about availability of computer code

Data collection	3D multiphoton imaging: Tiles were merged using Leica Application suite X (v3.3.0, Leica) with smooth overlap blending and data were visualized with Imaris software (v9.1, Bitplane).<br>3D lightsheet imaging: Three-dimensional reconstructions were obtained using Imaris imaging software (v9.1, Bitplane).<br>Histology imaging: Images were obtained using ZEN (v.2.1 Carl Zeiss)<br>The Drop-seq core computational pipeline was used for processing next generation sequencing reads of the scRNA-seq data, as previously described (Macosko et al., 2015). Briefly, STAR (version 2.5.2a) was used for mapping (Dobin et al., 2013).
Data analysis	All data represent the mean ± SEM. A Shapiro-Wilk's test (p>0.05) as well as visual inspection of the respective histograms, normal Q-Q plots and box plots were used to test whether samples were normally distributed (approximately), using IBM SPSS Statistics version 23. Two group comparisons were made using an unpaired Student's t-test for normally distributed data or a Mann–Whitney U test as the non-parametric equivalent. Comparisons between three or more groups were performed using a one-way ANOVA followed by Tukey's post hoc test for normally distributed data, or with a Kruskal-Wallis H test for non-normally distributed data. A value of p < 0.05 was considered statistically significant, where * is p < 0.05, ** is p < 0.01, and *** is p < 0.001. Analyses were performed with GraphPad Prism version 6 (GraphPad Software, Inc.). Directionality / Polar-coordinates plot was performed using the "ggplot2" library #1234 in R #5678 Version 3.4.1, 2.<br>To visualize the clustering result of the high dimensional single-cell data, the Fruchterman-Reingold algorithm from the Python toolkit Scanpy (v1.4.4.) was employed (Wolf et al., 2018). Additionally, to display the connectivity between the cell groups the partition based graph abstraction (PAGA) method was used (Wolf et al., 2018). The cells were grouped according to the time point of extraction. In the graph, those groups are represented as nodes and edges between the nodes show the connectivity or relatedness of these groups, therefore quantifying their similarity with respect to gene expression differences.<br>STAR (version 2.5.2a) was used for mapping (Dobin et al., 2013).<br>All image processing and analyses were performed with exported Tif images using Fiji (ImageJ 2.0.0 /1.52c, USA). Fluorescent channels were split and the brightness and contrast were adjusted to reduce background in order to prevent misinterpretation of background as cellular structures in the segmentation step.

LAS X (v3.4.218368),
Seurat Version 2.3.0
Excel (v16.0.12624.20422)

For manuscripts utilizing custom algorithms or software that are central to the research but not yet described in published literature, software must be made available to editors and reviewers. We strongly encourage code deposition in a community repository (e.g. GitHub). See the Nature Research guidelines for submitting code & software for further information.

Data

Policy information about availability of data

All manuscripts must include a data availability statement. This statement should provide the following information, where applicable:
- Accession codes, unique identifiers, or web links for publicly available datasets
- A list of figures that have associated raw data
- A description of any restrictions on data availability

All the data supporting the findings of this study are available within this paper and its supplementary information. ScRNAseq files are available at: https://www.ncbi.nlm.nih.gov/geo/ with the accession code: GSE149583

Field-specific reporting

Please select the one below that is the best fit for your research. If you are not sure, read the appropriate sections before making your selection.

☒ Life sciences ☐ Behavioural & social sciences ☐ Ecological, evolutionary & environmental sciences

For a reference copy of the document with all sections, see nature.com/documents/nr-reporting-summary-flat.pdf

Life sciences study design

All studies must disclose on these points even when the disclosure is negative.

Sample size	No statistical method was used to predetermine sample size. Required experimental sample sizes were estimated based on previous established protocols in the field. Experiments were independently repeated or carried out in biological replicates. The exact sample size (n) for each experimental group/condition was provided, and mean/the standard error were calculated in the manuscript.
Data exclusions	Not data were excluded from the analysis.
Replication	All attempts at replication were performed at least three times and were all successful. Experiments were carried out in biological replicates as indicated in the manuscript.
Randomization	Animals used in this study were randomised into various treatment groups based on a random numbers generator (www.random.org). In vitro experiments were also randomly assigned to treatments and conditions using a random numbers generator.
Blinding	Images used for processing and visual scoring were manually blinded before analysis. Other experiments presented in this study not required blinding.

Reporting for specific materials, systems and methods

We require information from authors about some types of materials, experimental systems and methods used in many studies. Here, indicate whether each material, system or method listed is relevant to your study. If you are not sure if a list item applies to your research, read the appropriate section before selecting a response.

Materials & experimental systems

n/a	Involved in the study	
☐	☒	Antibodies
☐	☒	Eukaryotic cell lines
☒	☐	Palaeontology and archaeology
☐	☒	Animals and other organisms
☐	☒	Human research participants
☒	☐	Clinical data
☒	☐	Dual use research of concern

Methods

n/a	Involved in the study	
☒	☐	ChIP-seq
☒	☐	Flow cytometry
☒	☐	MRI-based neuroimaging

Antibodies

Antibodies used	PDPN (mouse) Abcam, #ab11936 1:500<br>PDPN (human) Abcam, #ab10288 1:500

MSLN ABBIOTEC, #250519 1:200
HIF-1α Abcam, #ab179483) 1:200
CD44 polyclonal Abcam, #ab157107 1:200
CD44 monoclonal Abcam, #ab119348 1:200
Fibronectin Abcam, #ab23750 1:50
Collagen-3α1 Abcam, #ab7778 1:100
α-SMA Abcam, #ab21027 1:200
WT1 Abcam, #ab89901 1:200
GAPDH New England Biolabs, #2118S 1:2000
phosphoMYL9 Cell Signaling Technology, #3674 1:200
Cofilin Abcam, #ab42824 1:200
Ezrin Abcam, #ab4069 1:200
ARFGAP1 Abcam, #ab204405 1:200
pan Rho Abcam, #ab40673 1:200
α-Actinin Abcam, #ab18061 1:200
AKAP12 Invitrogen, #PA552281 1:200
ARP2 Santa Cruz, #sc-376698 1:200
ARP3 Santa Cruz, #sc-48344 1:200

Anti-N-cadherin Bio X Cell, #BE0184
Anti-CD106 / VCAM Bio X Cell, #BE0027
Anti-CD18 / Integrin β-2 Bio X Cell, #BE0009
Anti-CD54 / ICAM-1 Bio X Cell, #BE0020-1
Anti-CD44 Bio X Cell, #BE0039
Anti-CD62 / E-selectin Bio X Cell, #BE0294

Validation	See manufacturers' notes. Antibodies were additionally validated using respective isotype antibodies in immunofluorescence assays.

Eukaryotic cell lines

Policy information about cell lines

Cell line source(s)	Met-5A (ATCC CRL-9444)
Authentication	Stainings for mesothelial markers where successfully performed.
Mycoplasma contamination	All cells where tested negatively for mycoplasma contamination.
Commonly misidentified lines (See ICLAC register)	No commonly misidentified cell lines were used in the study.

Animals and other organisms

Policy information about studies involving animals; ARRIVE guidelines recommended for reporting animal research

Laboratory animals	All animal experiments were conducted under strict governmental and European guidelines and were approved by the local government for the administrative region of Upper Bavaria, under license number 55.2-1-54-2532-150-2015. Pathogen-free male and female C57BL/6 mice (6-10 week old) were obtained from Charles River and group-housed in climate-controlled quarters with a 12h/12h light/dark cycle. The animal rooms of the barriers are fully air-conditioned, the set points are in accordance with Annex A of the European Convention 2007/526 EC set to 20-24°C temperature and 45-65% humidity. Animals were allowed food and water ad libitum.<br>Rosa26mTmG or Rosa26tm1(DTA)Lky crossed with ProcrCreERT2-IRES-tdTomato pathogen-free male and female mice were group-housed in climate-controlled quarters with a 12h/12h light/dark cycle. The animal rooms of the barriers are fully air-conditioned, the set points are in accordance with Annex A of the European Convention 2007/526 EC set to 20-24°C temperature and 45-65% humidity. Animals were allowed food and water ad libitum. For lineage tracing experiments induced in 6-8 week adult mice, animals received three intraperitoneal injections of (Z)-4-Hydrotamoxifen (Sigma Aldrich, #H7904, 2 mg per 25 g body weight, diluted in 100 µL corn oil (Sigma Aldrich, #C8267)) every other day to induce activation of Cre recombinase.
Wild animals	The study did not involve wild animals.
Field-collected samples	The study did not involve samples collected from field.
Ethics oversight	All animal experiments were conducted under strict governmental and European guidelines and were approved by the local government for the administrative region of Upper Bavaria, under license number 55.2-1-54-2532-150-2015.

Note that full information on the approval of the study protocol must also be provided in the manuscript.

Human research participants

Policy information about studies involving human research participants

Population characteristics	Healthy donors between 55 - 75 years of age, both genders, underwent abdominal surgeries.
Recruitment	Adhesions were intraoperatively diagnosed and dissected from the respective organs and prepared for further analysis.Fresh human adhesions, from the abdomen, were collected through the Department of Chirurgische Klinik und Poliklinik, Klinikum rechts der Isar, Technical University Munich. Informed consent was obtained from all subjects after surgery. Participants were recruited randomly.
Ethics oversight	Local ethics committee of the Technical University of Munich, Germany (Nr. 173/18 S).

Note that full information on the approval of the study protocol must also be provided in the manuscript.
